# Ultra-Processed Food Consumption and Chronic Non-Communicable Diseases-Related Dietary Nutrient Profile in the UK (2008–2014)

**DOI:** 10.3390/nu10050587

**Published:** 2018-05-09

**Authors:** Fernanda Rauber, Maria Laura da Costa Louzada, Eurídice Martínez Steele, Christopher Millett, Carlos Augusto Monteiro, Renata Bertazzi Levy

**Affiliations:** 1 Center for Epidemiological Research in Nutrition and Health, University of São Paulo, São Paulo 01246-904, Brazil; maria.laura.louzada@gmail.com (M.L.d.C.L.); emar_steele@hotmail.com (E.M.S.); c.millett@imperial.ac.uk (C.M.); carlosam@usp.br (C.A.M.); rlevy@usp.br (R.B.L.); 2 Department of Nutrition, School of Public Health, University of São Paulo, São Paulo 01246-904, Brazil; 3 Department of Public Policies and Public Health, Federal University of São Paulo, Santos 11015-020, Brazil; 4 Public Health Policy Evaluation Unit, School of Public Health, Imperial College London, London W6 8RP, UK; 5 Department of Preventive Medicine, School of Medicine, University of São Paulo, São Paulo 01246-903, Brazil

**Keywords:** food processing, ultra-processed, diet quality, United Kingdom

## Abstract

We described the contribution of ultra-processed foods in the U.K. diet and its association with the overall dietary content of nutrients known to affect the risk of chronic non-communicable diseases (NCDs). Cross-sectional data from the U.K. National Diet and Nutrition Survey (2008–2014) were analysed. Food items collected using a four-day food diary were classified according to the NOVA system. The average energy intake was 1764 kcal/day, with 30.1% of calories coming from unprocessed or minimally processed foods, 4.2% from culinary ingredients, 8.8% from processed foods, and 56.8% from ultra-processed foods. As the ultra-processed food consumption increased, the dietary content of carbohydrates, free sugars, total fats, saturated fats, and sodium increased significantly while the content of protein, fibre, and potassium decreased. Increased ultra-processed food consumption had a remarkable effect on average content of free sugars, which increased from 9.9% to 15.4% of total energy from the first to the last quintile. The prevalence of people exceeding the upper limits recommended for free sugars and sodium increased by 85% and 55%, respectively, from the lowest to the highest ultra-processed food quintile. Decreasing the dietary share of ultra-processed foods may substantially improve the nutritional quality of diets and contribute to the prevention of diet-related NCDs.

## 1. Introduction

The prevalence of obesity and other diet-related chronic non-communicable diseases (NCDs), such as type II diabetes, hypertension, and some common cancers, is increasing worldwide [1]. At the same time, ultra-processed food production and consumption are steadily increasing in both high-income and lower-income countries [2,3,4,5,6]. Ultra-processed foods undermine food systems and dietary patterns based on minimally processed foods and freshly prepared meals [7,8].

Ultra-processed foods, as defined by the NOVA food classification system, are not modified or merely processed foods. They are industrial formulations manufactured from substances derived from foods, which typically contain cosmetic and various other types of additive and little if any intact food [9]. These products are designed to be extremely palatable and convenient, are often sold in large portion sizes, and are aggressively marketed [7,10].

Analyses of nationally representative dietary surveys conducted in the United States [11], Canada [12], and Brazil [13] consistently show that high consumption of ultra-processed foods renders nutritionally unbalanced diets. Moreover, a growing body of evidence suggests that such foods are harmful to health. Cross-sectional studies associate consumption of ultra-processed foods with obesity and related diseases [14,15,16,17]. A recent ecological study conducted with nationally representative data from nineteen European countries, including the U.K., found a direct association between household availability of ultra-processed foods and prevalence of obesity [18]. In prospective cohort studies, ultra-processed food consumption has been associated with incidence of obesity [19], hypertension [20], dyslipidaemias [21], and breast and total cancer [22].

To date, two studies on ultra-processed food consumption have already been conducted in the U.K. [23,24], both using an earlier version of NOVA that grouped processed foods together with ultra-processed foods. The first study on data collected by the 2008 Living Cost and Food Survey found that 63.4% of total dietary energy purchased by U.K. households came from processed or ultra-processed foods [23]. The second study on data from the 2008–2012 U.K. National Diet and Nutrition Survey (NDNS) estimated the average daily intake of processed or ultra-processed foods to be 53.1%, and showed an association between higher intakes of these foods and increased content of sodium, fat, saturated fat, carbohydrate, and sugar in the diet but not increased frequency of obesity [24].

The present study builds on these previous studies of ultra-processed food consumption in the U.K. First, we estimated the specific intake of ultra-processed foods using the current version of the NOVA classification [9]. Second, we enlarged the study period to 2008–2014 by adding the most recent NDNS available data set. Third, we examined associations between consumption of ultra-processed foods and nutrient intake recommended in international guidelines for the prevention of NCDs [25].

## 2. Materials and Methods

### 2.1. Data Source and Collection

We used data from the National Diet and Nutrition Survey Rolling Programme (NDNS) years 1–6 (2008/2009–2013/2014) combined, which is a cross-sectional survey of people aged 1.5 years and above. The survey was designed to be representative of the U.K. population and provides comprehensive information on food intake. Details of the rationale, design, and methods of the survey have been described in detail elsewhere [26]. Briefly, the sample was drawn from households randomly selected from the U.K. Postcode Address File, which is a list of all U.K. addresses. One adult (aged 19 years and over) and one child (aged 1.5–18 years), when available, were randomly selected from each household. From some households, only a child was selected to ensure approximately equal numbers of children and adults. Participants (or in the case of younger children, their parent/carer) completed a four-day food diary and participated in an interview to collect background data that included data on sociodemographic status.

Participants were asked to report all foods and drinks consumed both within and outside the home. Portion sizes were estimated using household measures or weights from packaging. Once completed, diaries were checked by interviewers with respondents and missing details added to improve completeness. Diary days were randomly selected to ensure balanced representation of all days of the week. All those who completed three or four days of dietary recording were included in the dataset, giving a sample size of 9374 (4738 adults and 4636 children) participants for years 1–6 combined.

The food intake data from completed records were coded and edited using the software DINO (Diet In, Nutrients Out) and food and nutrient intakes estimated using nutrient composition data from the Department of Health’s Nutrient Databank, updated for each survey year [27,28]. Our outcome measures are based on nutrient intake recommended by the World Health Organization (WHO) for prevention of NCDs: proteins, carbohydrates, free sugars, total fats, saturated fats, dietary fibre, sodium, and potassium [25,27,28,29,30,31,32]. Indicators relating to fibre, sodium, and potassium intake were expressed per 1000 kcal, while remaining nutrients were expressed as a percentage of total energy intake. Free sugars were calculated as non-milk extrinsic sugars (NMES) within the database [33,34]. The U.K. NDNS collected data on NMES and captured all free sugars, including those present in dried, stewed, or canned fruit. Dietary fibre intake was defined as non-starch polysaccharides as measured by the Englyst method [35].

Computerized raw data files and documentation from this survey were obtained under license from the U.K. Data Archive (http://www.esds.ac.uk). All relevant research ethics and governance committees approved the survey.

### 2.2. Food Classification According to Processing

We classified all recorded food items according to NOVA, a food classification system based on the nature, extent, and purpose of industrial food processing [9]. This classification includes four groups: unprocessed foods or minimally processed foods; processed culinary ingredients; processed foods; and ultra-processed foods. A detailed description of the NOVA classification can be found elsewhere [9,36].

All foods in NDNS are coded and grouped into subsidiary food groups (*n* = 155). When possible, subsidiary food groups were directly classified according to NOVA (see Supplementary Table S1). When foods within a subsidiary food group belonged to different NOVA groups (*n* = 52), the food codes were individually classified. Thus, we were able to classify each underlying ingredient of homemade dishes in the corresponding NOVA group.

Most food items in the NDNS were systematically disaggregated into their individual components, but about 4% of composite food codes were still mixed dishes compiled from two or more single-ingredient food codes [37]. Using the core sample of years 1–4 (*n* = 4125), we estimated that these represented only 3% of total dietary energy. In these cases, dishes were categorised according to the main constituent ingredient. Dishes in which a main constituent ingredient was not clearly identified (for example, chicken and vegetable soup) were classified as a specific subgroup of freshly prepared dishes based on one or more unprocessed or minimally processed food (group 1). Non-caloric supplements were not included in the analyses.

### 2.3. Covariates

Covariates included were age in years, sex, ethnicity (white, mixed ethnic group, black or black British, Asian or Asian British, and other race). Household income was equivalised for different household sizes and composition using the McClements scale [26]. When values for the equivalised household income were missing (12.8%), we used multiple imputation by the chained equation method based on age, sex, race/ethnicity, and ultra-processed food consumption. This was done 20 times. The Monte Carlo error analysis showed good statistical reproducibility of the results [38].

### 2.4. Data Analysis

For each survey day and age group, we defined extreme total dietary energy intake outliers as values above the 99th or below the 1st percentiles [39]. Based on these criteria, we excluded 10 individuals who had all days of food diary classified as outliers. In total, 9364 (4729 adults and 4635 children) participants were eligible for inclusion in the analyses. More than 91% completed the four food diary days. We used the mean of all available days of food diary for each person.

First, we estimated the distribution of total dietary energy intake according to NOVA food groups and subgroups. Then, we examined how the energy share of each NOVA food group and subgroup varied across quintiles of the energy share provided by ultra-processed foods. We also estimated nutritional indicators of the overall diet for the whole population and across quintiles of the dietary energy share of ultra-processed foods. Linear regression models were used to test trends across quintiles of the dietary energy share provided by ultra-processed foods. Standardized regression coefficients (β) were estimated to allow for comparisons across variables with different units.

Finally, using the recommended dietary nutrient goals for the prevention of chronic diseases specified by the WHO [25,30,32], we evaluated the prevalence of inadequate intake of free sugars (≥10% of total energy), saturated fats (≥10% of total energy), fibre (<10 g/1000 kcal), sodium (≥1 g/1000 kcal), and potassium (<1755 mg/1000 kcal) across quintiles of the energy share provided by ultra-processed foods. Prevalence ratios were estimated using Poisson regression.

Adjusted regression models were also performed to control for age (years), sex, ethnicity, and equivalised household income (in pounds sterling). NDNS study weights were used in all analyses to account for sampling and non-response error. All statistical analyses were carried out using Stata Statistical Software version 14. The p values reported were two-tailed, and a p value of <0.01 was considered statistically significant.

## 3. Results

### 3.1. Distribution of Total Energy Intake According to NOVA Food Groups and across Quintiles of Dietary Share of Ultra-Processed Foods

The population mean energy intake was 1764 kcal/day, with 30.1% of dietary energy coming from unprocessed or minimally processed foods, 4.2% from processed culinary ingredients, 8.8% from processed foods, and 56.8% from ultra-processed foods (Table 1). The most common unprocessed or minimally processed foods in terms of dietary energy were milk and plain yogurt (5%), potatoes and other tubers and roots (3.5%), fruits (3.3%), and red meat (3.3%). Table sugar (1.7%) and butter (1.5%) provided the highest percentage of dietary energy among processed culinary ingredients. Beer and wine (3.5%) and cheese (2.9%) provided the highest percentage of dietary energy among processed foods. Industrialised packaged breads (11%), packaged pre-prepared meals (7.7%), breakfast cereals (4.4%), sausage and other reconstituted meat products (3.8%), confectionery (3.5%), biscuits (3.5%), pastries, buns, and cakes (3.3%), industrial chips (French fries) (2.8%), and soft and fruit drinks (2.5%) provided the highest percentage of dietary energy among ultra-processed foods.

The mean dietary share of ultra-processed foods ranged from around one third (34.9%) of total calories (1st quintile) to almost 80% (78.0%) (5th quintile). The dietary share of all subgroups of ultra-processed foods increased across quintiles except for sauces, dressing, and gravies (Table 2). From the lowest to the highest ultra-processed food quintile, the greatest increases in dietary share came from industrial pizza (+630%), soft and fruit drinks (+410%), industrial chips (French fries) (+319%), and packaged salty snacks (+257%). The dietary share of all three remaining NOVA food groups (unprocessed or minimally processed foods, processed culinary ingredients, and processed foods) decreased across quintiles of ultra-processed food consumption.

### 3.2. Dietary Nutrient Profile Indicators

Table 3 shows the mean content of selected nutrients in the U.K. diet overall and across quintiles of the energy share from ultra-processed foods. The overall mean intake of free sugars, total fats, saturated fats, and sodium exceeds the maximum recommended WHO values while mean dietary fibre and potassium intake are much lower than the minimum recommended values.

As the contribution of ultra-processed foods to total energy intake increased, the dietary contents of carbohydrates, free sugars, total fats, saturated fats, and sodium increased uniformly and significantly while the dietary content of proteins, fibre, and potassium decreased. For example, as stated, free sugar intake increased from 9.9% to 15.4% of total energy from the first to the last quintile. Except for saturated fats, all trends remained significant after adjustment for sociodemographic covariates.

The prevalence of inadequate nutrient intake across quintiles of the energy share of ultra-processed foods is shown in Table 4. A large majority of the U.K. population did not meet the WHO recommended intake for free sugars, saturated fats, fibre, sodium, and potassium. As the contribution of ultra-processed foods to total energy intake increased, the prevalence of inadequate nutrient intake of free sugars, saturated fat, fibre, sodium, and potassium increased significantly. Most notable was the trend of free sugars and sodium, which increased by 85% and 55%, respectively, from the lowest to the highest ultra-processed food quintile (free sugars from 41.9% to 77.2% and sodium from 55.8% to 86.7%).

## 4. Discussion

In this analysis of nationally representative data, we found that more than half of the dietary energy consumed on average by the U.K. population came from ultra-processed foods, notably industrialised packaged breads, packaged pre-prepared meals, breakfast cereals, reconstituted meat products, confectionery, biscuits, pastries, buns and cakes, industrial chips (French fries), and soft drinks, fruit drinks, and fruit juices.

We also found a significant linear inverse association between the dietary contribution of ultra-processed foods and the dietary content of protein, fibre, and potassium. Carbohydrates, free sugars, total fats, saturated fats, and sodium contents increased significantly as ultra-processed food consumption increased. Thus, our findings show strong associations between consumption of ultra-processed foods and dietary nutrient profiles that predict increased risk of several diet-related NCDs [25,30,32].

The high dietary share of ultra-processed foods in the U.K. is similar to that found in other countries, such as the United States (58% of total energy) [11] and Canada (48% of total energy) [12]. It is currently much greater than in some other high-income countries, such as France (35.9% of total energy) [16], and middle-income countries, such as Brazil (20.4% of total energy) [14], where traditional diets based on freshly prepared meals still prevail.

The negative impact of ultra-processed food consumption on the overall dietary quality observed in our study is consistent with results from the United States [11], Canada [12], and Brazil [13,40]. An exception is the positive association between the dietary share of ultra-processed foods and sodium content, which has not been observed in the United States, Canada, and Brazil. This may be because of differences in the type of ultra-processed foods consumed in each country, with salted products more likely to be consumed in the U.K. population than the sweetened products more typically consumed in the United States [11] and Canada [12]. Increases in sodium intake across quintiles of ultra-processed food consumption are likely specifically to reflect increased consumption of industrial pizza (which as mentioned increased more than 600% from the lowest to the highest quintile of the dietary share of ultra-processed foods), chips (French fries), packaged salty snacks, and packaged pre-prepared meals.

An important finding of our study is that more than half of the U.K. population was not within the range of values recommended by the WHO for the prevention of NCDs, and that these proportions strongly increased with the rise of the share of ultra-processed foods in the diet. In the higher quintile of ultra-processed food consumption, about 80% of the U.K. population exceeded the upper limits recommended by the WHO for free sugars, saturated fats, and sodium and over 90% did not meet the recommendation for dietary fibre and potassium.

The poor nutritional quality of ultra-processed foods coupled with their high availability, low cost, and aggressive marketing, which result in excessive consumption [23,41], can lead to obesity [14,18] and other chronic diet-related NCDs [17,21]. In Spain, the association between the consumption of ultra-processed foods and the 9-year incidence of obesity [19] and hypertension [20] was found in a large cohort of adults. A large prospective cohort study conducted with French adults showed that a 10% increase in the proportion of ultra-processed foods in the diet was associated with a 12% and 11% significant increase in overall and breast cancer risks, respectively [22]. Lastly, a modelling exercise based on the nutrient profile of ultra-processed foods consumed in the U.K. has shown that reducing consumption of ultra-processed foods could prevent or postpone approximately 10% of cardiovascular deaths [42]. For all these reasons, replacing ultra-processed foods by unprocessed or minimally processed foods and dishes made with these foods seems essential to ensure healthy diets in the U.K.

There are some specific limitations of this study that warrant discussion. The data we used was self-reported and may be subject to social desirability bias. A limitation of dietary assessment methods is underreporting of some foods (particularly unhealthy foods), though food diaries are recognised to be one of the most comprehensive methods of assessing dietary intake. A possible underreporting of unhealthy foods may lead to an underestimation of the dietary contribution of ultra-processed foods and the overall intake of some nutrients (such as free sugars, sodium, and saturated fat), but does not necessarily affect the relationship between the dietary share of ultra-processed foods and the overall dietary nutrient profile. Nevertheless, accurate and valid NDNS data were achieved through optimal methods for collecting dietary intake [43], which helped to minimise misreporting. Although NDNS collects limited information indicative of food processing (for example, place of meals and product brands), these data are not consistently determined for all food items, which can lead to misclassification of food items. This bias is more likely for a few particular food items that have insufficient information, such as pizza and specific dishes (see Supplementary Table S1). In those cases, the most frequently consumed alternative (culinary preparation or manufactured product) was chosen. Despite these limitations, this study has a number of key strengths. We used data from the most recent and available population-based survey and applied weighting to reduce any sampling and non-response bias. Thereby, our results are probably generalizable to the U.K. population and may also be applicable to similar international contexts. Unlike household budget data, food diaries take food wastage into account, include unpackaged food and food eaten out of home, and do not assume that all individuals within a household consume the same diet. The NDNS Nutrient Databank is updated annually by Public Health England. Updating of the databank includes the addition of new foods as well as revision of nutrient composition of existing foods or to take account of reformulation by manufacturers and changes in fortification practices. This is particularly important for findings related to sodium content considering current U.K. government actions to encourage the food industry to reduce salt in manufactured foods. Finally, we used the NOVA system that has been recognized as a tool in nutrition research with potential for wider application in food policy [44].

The average consumption of ultra-processed foods in the U.K. is alarmingly high, reflecting the ubiquity of these products in the food environment and their aggressive advertising and promotion [5,10]. Our finding that even in the lowest quintile of ultra-processed food consumption the majority of participants did not meet WHO recommendations for most nutrient intakes has important policy implications for the U.K.. It suggests that behavioural interventions are unlikely to substantially shift the population mean toward a healthier nutrient profile. This assertion is supported by a study of British adults that showed that better home food preparation skills and more frequent use of these skills was associated with only modest reductions in lower ultra-processed foods consumption [45]. More radical whole population strategies are needed to achieve necessary reductions in ultra-processed food consumption, including taxation and pricing interventions, adequate food labelling, and restrictions on advertising and promotion.

## 5. Conclusions

Reducing the dietary share of ultra-processed foods by increasing the consumption of unprocessed or minimally processed foods and freshly prepared dishes and meals made from these foods can be an effective way to improve substantially the nutritional quality of diets in the U.K. and contribute to the prevention of obesity and other chronic diet-related NCDs. However, given the ubiquity of ultra-processed foods in U.K. society, radical whole population strategies will be needed to achieve necessary reductions in ultra-processed food consumption.

## Figures and Tables

**Table 1:** Distribution of total energy intake according to NOVA food groups. U.K. population aged 1.5 years or over (2008–2014).

NOVA Food Groups ^a^	Mean Absolute Intake (Kcal/Day)		Mean Relative Intake (% of Total Energy Intake)	
	Mean	SE	Mean	SE
Unprocessed or minimally processed foods	514.29	3.84	30.15	0.21
Milk and plain yoghurt	82.92	1.06	4.97	0.06
Potatoes and other tubers and roots	59.86	0.96	3.50	0.06
Fruits	54.94	0.98	3.33	0.06
Red meat	56.96	1.16	3.29	0.07
Poultry	46.03	1.02	2.70	0.06
Cereals ^b^	42.74	1.44	2.52	0.08
Pasta	30.76	0.90	1.80	0.05
Eggs	24.88	0.59	1.46	0.03
Vegetables	25.84	0.45	1.52	0.03
Fresh fruit juice ^c^	20.78	0.64	1.21	0.04
Fish	21.34	0.73	1.22	0.04
Legumes	10.57	0.39	0.63	0.02
Other unprocessed or minimally processed foods ^d^	36.67	1.01	2.05	0.06
**Processed culinary ingredients**	**75.18**	**1.47**	**4.20**	**0.08**
Table sugar	29.36	0.90	1.66	0.05
Butter ^e^	27.12	0.84	1.53	0.05
Plant oil	12.18	0.54	0.69	0.03
Other processed culinary ingredients ^f^	6.51	0.42	0.34	0.02
**Processed foods**	**167.33**	**2.88**	**8.83**	**0.13**
Beer and wine	70.70	2.30	3.54	0.11
Cheese	51.32	1.12	2.86	0.06
Vegetables and other plant foods preserved in brine	16.58	0.42	0.95	0.02
Processed breads	13.67	0.90	0.72	0.04
Ham and other salted, smoked, or canned meat or fish	9.53	0.43	0.54	0.02
Other processed foods ^g^	5.52	0.41	0.30	0.02
**Ultra-processed foods**	**996.53**	**6.62**	**56.82**	**0.24**
Industrialised packaged breads	191.16	2.00	11.01	0.10
Packaged pre-prepared meals ^h^	131.95	2.19	7.66	0.12
Breakfast cereals	73.51	1.33	4.36	0.07
Sausage and other reconstituted meat products	67.12	1.19	3.84	0.06
Confectionery	64.82	1.42	3.55	0.07
Biscuits	60.35	1.21	3.46	0.06
Pastries, buns, and cakes	59.54	1.43	3.26	0.07
Industrial chips (French fries)	48.55	1.14	2.79	0.06
Soft drinks, fruit drinks, and fruit juices	45.77	1.28	2.49	0.06
Milk-based drinks	37.52	0.86	2.23	0.05
Packaged salty snacks	36.24	0.84	2.02	0.04
Industrial pizza	33.62	1.38	1.84	0.07
Margarine and other spreads	38.49	0.76	2.19	0.04
Sauces, dressing, and gravies	37.62	0.75	2.11	0.04
Industrial desserts	15.53	0.62	0.87	0.03
Other ultra-processed foods ^i^	54.75	1.43	3.08	0.08

^a^ Some items may include culinary preparations with oil, fats, salt, and sugar; ^b^ Including grains and flours; ^c^ Including ultra-high temperature processing (UHT) or pasteurized, and smoothies; ^d^ Including coffee, tea, sea foods, fungi, nuts, and freshly prepared dishes based on one or more unprocessed or minimally processed food; ^e^ Including lard and suet shredded; ^f^ Including starches, coconut and milk cream, gelatin powder, and vinegar; ^g^ Including salted, sweetened, or oil-roasted nuts or seeds, condensed milk, and commercial baby foods; ^h^ Including frozen and shelf-stable dishes and canned soups; ^i^ Including baked beans, meat alternatives, soy and other drinks as milk substitutes, infant formula, and distilled alcoholic drink. SE = standard error.

**Table 2:** Distribution (%) of total energy intake according to NOVA food groups across strata of increasing ultra-processed food consumption. U.K. population aged 1.5 years or over (2008–2014).

	Mean Relative Intake (% of Total Energy Intake)				
NOVA Food Groups ^a^	Quintiles of the Contribution of Ultra-Processed Foods to Total Energy Intake				
	Q1	Q2	Q3	Q4	Q5
**Unprocessed or minimally processed foods**	**44.73**	**35.16**	**30.11**	**24.81**	15.95 *
Milk and plain yoghurt	5.76	5.59	5.11	4.75	3.64 *
Potatoes and other tubers and roots	4.40	4.02	3.85	3.08	2.17 *
Fruits	5.23	3.73	3.36	2.62	1.74 *
Red meat	5.05	4.13	3.28	2.53	1.48 *
Poultry	3.89	3.16	2.67	2.33	1.46 *
Cereals ^b^	5.57	2.69	2.12	1.44	0.77 *
Pasta	2.59	2.18	1.66	1.53	1.02 *
Eggs	2.03	1.70	1.45	1.29	0.82 *
Vegetables	2.48	1.81	1.50	1.21	0.61 *
Fresh fruit juice ^c^	1.43	1.29	1.34	1.15	0.82 *
Fish	2.11	1.45	1.28	0.86	0.42 *
Legumes	1.06	0.76	0.65	0.44	0.23 *
Other unprocessed or minimally processed foods ^d^	3.22	2.70	1.88	1.61	0.82 *
**Processed culinary ingredients**	**6.49**	**5.08**	**3.93**	**3.18**	2.30 *
Table sugar	2.10	1.84	1.55	1.51	1.28 *
Butter ^e^	2.19	2.07	1.55	1.12	0.73 *
Plant oil	1.58	0.80	0.52	0.37	0.19 *
Other processed culinary ingredients ^f^	0.67	0.40	0.33	0.19	0.11 *
**Processed foods**	**13.90**	**11.02**	**8.91**	**6.65**	3.69 *
Beer and wine	6.88	4.62	3.26	2.06	0.89 *
Cheese	3.42	3.41	3.20	2.66	1.62 *
Vegetables and other plant foods preserved in brine	1.31	1.12	1.00	0.81	0.54 *
Processed breads	1.06	1.04	0.61	0.56	0.32 *
Ham and other salted, smoked, or canned meat or fish	0.92	0.59	0.63	0.35	0.23 *
Other processed foods ^g^	0.42	0.32	0.32	0.27	0.15 *
**Ultra-processed foods**	**34.89**	**48.74**	**57.06**	**65.37**	78.06 *
Industrialised packaged breads	8.43	10.87	11.60	11.87	12.27 *
Packaged pre-prepared meals ^h^	3.78	5.57	7.19	9.29	12.46 *
Breakfast cereals	3.58	4.43	4.69	4.87	4.21 *
Sausage and other reconstituted meat products	2.57	3.51	3.94	4.09	5.08 *
Confectionery	1.67	2.60	3.37	4.38	5.71 *
Biscuits	2.28	2.89	3.48	4.21	4.44 *
Pastries, buns, and cakes	1.99	2.92	3.51	3.87	4.00 *
Industrial chips (French fries)	1.21	1.73	2.59	3.35	5.07 *
Soft drinks, fruit drinks, and fruit juices	0.94	1.62	2.21	2.86	4.82 *
Milk-based drinks	1.40	2.15	2.38	2.41	2.80 *
Packaged salty snacks	0.95	1.44	1.81	2.50	3.41 *
Industrial pizza	0.50	1.10	1.67	2.29	3.65 *
Margarine and other spreads	1.31	2.02	2.27	2.51	2.83 *
Sauces, dressing, and gravies	2.02	2.30	2.20	2.24	1.82
Industrial desserts	0.50	0.81	1.00	0.93	1.10 *
Other ultra-processed foods ^i^	1.58	2.56	3.01	3.51	4.72 *
**Total**	**100**	**100**	**100**	**100**	**100**

^a–i^ See Table 1 footnote. * *p* < 0.001 for linear trend across quintiles of ultra-processed food consumption.

**Table 3:** Nutritional indicators of the overall diet across strata of increasing ultra-processed food consumption. U.K. population aged 1.5 years or over (2008–2014).

Indicator ^a,b^	Overall Diet		Quintiles of the Contribution of Ultra-Processed Foods to Total Energy Intake					Standardized Regression Coefficient	
	*Mean*	*SE*	Q1	Q2	Q3	Q4	Q5	Crude	Adjusted ^c^
Energy intake (kcal/d)	1764.71	8.55	1732.00	1766.35	1784.89	1776.38	1763.96	0.02	0.03
Percentage of energy intake from:									
Proteins	15.77	0.06	17.24	16.57	16.00	15.19	13.84	-0.32 *	-0.30 *
Carbohydrates	48.65	0.12	45.45	47.27	48.61	50.07	51.84	0.30 *	0.26 *
Free sugars	12.44	0.10	9.94	11.34	12.16	13.38	15.41	0.29 *	0.23 *
Fats	32.18	0.09	31.37	31.93	32.12	32.47	33.04	0.09 *	0.11 *
Saturated fats	12.10	0.05	11.67	12.19	12.24	12.18	12.19	0.04 *	0.03
Dietary fibre density (g/1000 kcal)	7.70	0.04	8.36	8.04	7.81	7.46	6.86	-0.20 *	-0.13 *
Sodium density (g/1000 kcal)	1213.91	5.11	1072.57	1204.21	1228.75	1245.29	1318.85	0.23 *	0.22 *
Potassium density (mg/1000 kcal)	1547.86	5.94	1735.20	1635.79	1571.38	1470.65	1326.19	-0.37 *	-0.31 *

^a^ All values refer to means; ^b^ Values recommended by the World Health Organization (WHO) for proteins (10–15% of total energy), carbohydrates (55–75% of total energy), free sugars (<10% of total energy), fats (15–30% of total energy), saturated fats (<10% of total energy), dietary fibre (≥10 g/1000 kcal), sodium (<1 g/1000 kcal), and potassium (≥1755 mg/1000 kcal); ^c^ Adjusted for age (years), sex, ethnicity (white, mixed ethnic group, black or black British, Asian or Asian British, and other race), and equivalised household income (in pounds sterling); * *p* < 0.01 for linear trend across quintiles of ultra-processed food consumption.

**Table 4:** Prevalence of inadequate nutrient intake across strata of increasing ultra-processed food consumption. UK population aged 1.5 years or over (2008–2014).

Indicator	Overall Diet	Quintiles of the Contribution of Ultra-Processed Foods to Total Energy Intake					PR	
		Q1	Q2	Q3	Q4	Q5	Crude	Adjusted ^a^
	Individuals who did not meet the recommendation (%)							
Free sugars (≥10% of total energy)	61.27	41.87	56.35	60.76	70.18	77.20	1.15 *	1.12 *
Saturated fats (≥10% of total energy)	74.45	64.00	73.60	77.16	77.26	80.24	1.05 *	1.04 *
Dietary fibre density (<10 g/1000 kcal)	84.02	73.69	80.33	83.77	88.75	93.59	1.06 *	1.04 *
Sodium density (≥1 g/1000 kcal)	74.92	55.79	73.54	78.11	80.49	86.69	1.10 *	1.09 *
Potassium density (<1755 mg/1000 kcal)	74.76	56.12	68.40	73.13	83.83	92.35	1.13 *	1.10 *

PR = Prevalence ratios estimated using Poisson regression; ^a^ Adjusted for age (years), sex, race/ethnicity (white, mixed ethnic group, black or black British, Asian or Asian British, and other race), and equivalised household income (in pounds sterling); * *p* < 0.001 for linear trend across quintiles of ultra-processed food consumption.

## Supplementary Materials

The following are available online at http://www.mdpi.com/2072-6643/10/5/587/s1, Table S1: Coding of subsidiary food groups from National Diet and Nutrition Survey according to NOVA classification.
