# Supplementary material for: Ultra-Processed Food Consumption and Chronic Non-Communicable Diseases-Related Dietary Nutrient Profile in the UK (2008–2014)
Source: Nutrients. 2018 May 9;10(5):587. doi: 10.3390/nu10050587 (PMC5986467; doi:10.3390/nu10050587)
Supplement: Supplementary file 1 [file nutrients-10-00587-s001.pdf]

Supplementary Table S1. Coding of subsidiary food groups from National Diet and Nutrition Survey according to NOVA classification.

Subsidiary food group code	Subsidiary food group name	NOVA food group†
1C	Pizza	4
1D	Pasta (manufactured products and ready meals	4
1E	Pasta (other, including homemade dishes)	*
1F	Rice (manufactured products and ready meals)	4
1G	Rice (other, including homemade dishes)	*
1R	Other cereals	*
2R	White bread (not high fibre, not multiseed bread)	4
3R	Wholemeal bread	4
4R	Other bread	4
5R	High fibre breakfast cereals	4
6R	Other breakfast cereals (not high fibre)	4
7A	Biscuits(manufactured/retail)	4
7B	Biscuits (homemade)	*
8B	Fruit pies (manufactured)	4
8C	Fruit pies (homemade)	*
8D	Buns cakes and pastries (manufactured)	4
8E	Buns cakes and pastries (homemade)	*
9C	Cereal based milk puddings (manufactured)	4
9D	Cereal based milk puddings (homemade)	*
9E	Sponge puddings (manufactured)	4
9F	Sponge puddings (homemade)	*
9G	Other cereal based puddings (manufactured)	4
9H	Other cereal based puddings (homemade)	*
10R	Whole milk	1
11R	Semi-skimmed milk	1
12R	Skimmed milk	1
13A	Infant formula	4
13B	Cream (including imitation cream)	*
13R	Other milk	*
14A	Cottage cheese	3
14B	Cheddar cheese	3
14R	Other cheese	*
15B	Yogurt	*
15C	Fromage frais and other dairy desserts (manufactured)	4
15D	Dairy desserts (homemade)	*
16C	Manufactured egg products, including ready meals	4
16D	Other eggs and egg dishes, including homemade	*
17R	Butter	2
18A	Polyunsaturated margarine	4
18B	Polyunsaturated oils	2
19A	Polyunsaturated low fat spread	4
19R	Low fat spread not polyunsaturated	4
20A	Block margarine	4
20B	Soft margarine not polyunsaturated	4
20C	Other cooking fats and oils not polyunsaturated	2
21A	Reduced fat spread (polyunsaturated)	4
21B	Reduced fat spread (not polyunsaturated)	4
22A	Ready meals/meal centres based on bacon and ham	4
22B	Other bacon and ham (including homemade dishes)	*
23A	Manufactured beef products (including ready meals)	4
23B	Other beef & veal (including homemade recipe dishes)	*
24A	Manufactured lamb products (including ready meals)	4
24B	Other lamb (including homemade recipe dishes)	*
25A	Manufactured pork products(including ready meals)	4
25B	Other pork (including homemade recipe dishes)	*
26A	Manufactured coated chicken/turkey products	4
27A	Manufactured chicken products (including ready meals)	4
27B	Other chicken/turkey (including homemade recipe dishes)	*
28R	Liver and dishes	*
29R	Burgers and kebabs purchased	4
30A	Ready meals based on sausages	4
30B	Other sausages (including homemade dishes)	*
31A	Meat pies and pastries (manufactured)	4
31B	Meat pies and pastries (homemade)	*
32A	Other meat products (manufactured including ready meals)	4
32B	Other meat (including homemade recipe dishes)	*
33R	White fish coated or fried	*
34C	Manufactured white fish products (including ready meals)	4
34D	Other white fish (including homemade dishes)	*
34E	Manufactured shellfish products (including ready meals)	4
34F	Other shellfish (including homemade dishes)	*
34G	Manufactured canned tuna products (including ready meals)	*

34H	Other canned tuna (including homemade dishes)	*
35A	Manufactured oily fish products (including ready meals)	4
35B	Other oily fish (including homemade dishes)	*
36A	Carrots (raw)	1
36B	Salad and other raw vegetables	*
36C	Tomatoes raw	1
37A	Peas not raw	*
37B	Green beans not raw	*
37C	Baked beans	4
37D	Leafy green vegetables not raw	*
37E	Carrots not raw	*
37F	Tomatoes not raw	*
37I	Beans and pulses (including ready meal & homemade dishes)	*
37K	Meat alternatives (including ready meals and homemade dishes)	4
37L	Other manufactured vegetable products (including ready meals)	4
37M	Other vegetables (including homemade dishes)	*
38A	Chips purchased including takeaway	4
38C	Other manufactured potato products fried/baked	4
38D	Other fried/roast potatoes (including homemade dishes)	*
39A	Other potato products and dishes(manufactured)	4
39B	Other potatoes (including homemade dishes)	*
40A	Apples and pears not canned	*
40B	Citrus fruit not canned	*
40C	Bananas	*
40D	Canned fruit in juice	*
40E	Canned fruit in syrup	3
40R	Other fruit not canned	*
41A	Sugar	*
41B	Preserves	3
41R	Sweet spreads fillings and icing	4
42R	Crisps and savoury snacks	4
43R	Sugar confectionery	4
44R	Chocolate confectionery	4
45R	Fruit juice	*
47A	Liqueurs	4
47B	Spirits	4
48A	Wine	3
48B	Fortified wine	4
48C	Low alcohol and alcohol free wine	3
49A	Beers and lagers	3
49B	Low alcohol & alcohol free beer & lager	3
49C	Cider and Perry	4
49D	Low alcohol & alcohol free cider & Perry	4
49E	Alcoholic soft drinks (Alcopops)	4
50A	Beverages dry weight	4
50C	Soup (manufactured/retail)	4
50D	Soup (homemade)	*
50E	Nutrition powders and drinks	4
50R	Savoury sauces pickles gravies & condiments	4
51A	Coffee (made up weight)	*
51B	Tea (made up)	*
51C	Herbal tea (made up)	1
51D	Bottled water still or carbonated	*
51R	Tap water only	1
52A	Commercial toddlers drinks	3
52R	Commercial toddlers foods	3
53R	Ice cream	4
54A	Cod liver oil and other fish oils	**
54B	Evening primrose oil and other plant oils	**
54C	Single vitamins/minerals not Folic acid, iron, calcium	**
54D	Folic acid	**
54E	Iron only or with vitamin C	**
54F	Calcium only or with vitamin D	**
54G	Vitamins (two or more including multivitamins) no minerals	**
54H	Minerals (two or more including multimineral) no vitamins	**
54I	Vitamins and minerals (including multivitamins & minerals)	**
54J	Non-nutrient supplements (including herbal)	**
54K	Other nutrient supplements	**
54L	Vitamin C	**
54M	Single vitamins/minerals not Folic acid, iron, calcium or vitamin C	**
54N	Cod liver oil and other fish oils (including with vitamins A, D, E)	**
54P	Multivitamins and/or minerals with omega ultra-processed	**
55R	Artificial sweeteners	4
56R	Nuts and seeds	*
57A	Soft drinks not low calorie concentrated	4
57B	Soft drinks not low calorie carbonated	4

57C	Soft drinks not low calorie, ready to drink, still	4
58A	Soft drinks low calorie concentrated	4
58B	Soft drinks low calorie carbonated	4
58C	Soft drinks low calorie, ready to drink, still	4
59R	Brown, granary and wheat germ bread	4
60R	1% Milk	1
61R	Smoothies	1

† NOVA food groups defined as 1) unprocessed or minimally processed foods; 2) processed culinary ingredients; 3) processed foods; and 4) ultra-processed foods.

* All foods within this subsidiary food group were individually coded (by food name).

** Supplements were not included in any of the NOVA food groups.
